# Metabolomic and transcriptomic exploration of the uric acid-reducing flavonoids biosynthetic pathways in the fruit of *Actinidia arguta Sieb. Zucc*.

**DOI:** 10.3389/fpls.2022.1025317

**Published:** 2022-10-27

**Authors:** Yubo Wang, Minghui Zhang, Kuiling Dong, Xiaojuan Yin, Chunhui Hao, Wenge Zhang, Muhammad Irfan, Lijing Chen, Yong Wang

**Affiliations:** ^1^ Key Laboratory of Agriculture Biotechnology, College of Biosciences and Biotechnology, Shenyang Agricultural University, Shenyang, China; ^2^ Oriental Language Institute, Mudanjiang Normal University, Mudanjiang, China; ^3^ Pet Medicine Teaching and Research Office, Liaoning Agricultural College, Yingkou, China; ^4^ Biochemistry Teaching and Research Office, Anshan Health School, Anshan, China; ^5^ Department of Biotechnology, University of Sargodha, Sargodha, Pakistan; ^6^ College of Chemical Engineering, Liaoning University of Science and Technology, Anshan, China

**Keywords:** flavonoids, *Actinidia arguta Sieb.Zucc.*, transcriptome, widely targeted metabonomics, biosynthetic pathway

## Abstract

Flavonoids from *Actinidia arguta Sieb. Zucc.* can reduce uric acid in mice. However, the molecular basis of its biosynthesis is still unclear. In this paper, we used a combination of extensively targeted metabolomics and transcriptomics analysis to determine the types and differences of flavonoids in the fruit ripening period (August to September) of two main cultivated varieties in northern China. The ethanol extract was prepared, and the potential flavonoids of Chrysin (Flavone1), Rutin (Flavone2), and Daidzein (Flavone3) in *Actinidia arguta Sieb. Zucc.* were separated and purified by HPD600 macroporous adsorption resin and preparative liquid chromatography. The structure was identified by MS-HPLC, and the serum uric acid index of male Kunming mice was determined by an animal model test.125 flavonoids and 50 differentially regulated genes were identified. The contents of UA (uric acid), BUN (urea nitrogen), Cr (creatinine), and GAPDH in mouse serum and mouse liver glycogen decreased or increased in varying degrees. This paper reveals the biosynthetic pathway of uric acid-reducing flavonoids in the fruit of *Actinidia arguta Sieb. Zucc.*, a major cultivar in northern China, provides valuable information for the development of food and drug homologous functional foods.

## Introduction

Flavonoids are one of the main secondary metabolites in Actinidia arguta Sieb. Zucc. Structurally, they are mainlyflavonols, dihydroflavonols, 3-o-flavonoid glycosides, and their derivatives. Wojdyło and Nowicka (2019) identified the polyphenol compounds in Actinidia arguta Sieb. Zucc. and obtained 16 flavonols, 7 flavonols, 7 phenolic acids, and 1 anthocyanin. To explore the relationship between metabolite changes and fruit color changes. Li et al. (2018b) carried out transcriptomics and metabolomics analysis on the flesh of two kinds of Actinidia arguta Sieb. Zucc. and identified a variety of flavonoids such as bitter bracteachin, luteolin, dihydromyricetin, anthocyanin, geranium, delphinidin, and (-) - epigallocatechin. Jang et al. (2009) isolated two new flavonoids with γ-lactams from the roots of Actinidia arguta Sieb. Zucc., which are flavan-3,6-(2-pyrrolidinome-5-yl)-(−)-epicatechin and 8-(2-phrrolidinone-5-yl)-(−)-epicatechin and also get proanthocyanidin B-4.Flavonoids have physiological functions such as antioxidant, antiviral, prevention and treatment of cardiovascular and cerebrovascular diseases, prevention of hyperuricemia, liver protection, and immunity (Latocha et al., 2013; Hu et al., 2016; Jiang et al., 2020).

Hyperuricemia (HUA) is a pathological state in which UA levels in the blood increase continuously or the blood is supersaturated with UA. The number of patients with HUA in China exceeded 17 million in 2017, and the data shows a rapid increase in cases, with an annual growth rate of 9.7%. Gender, age, race, and lifestyle habits all affect the incidence of HUA (Lanaspa et al., 2011; Chen et al., 2022; Maruhashi et al., 2022; Tsai et al., 2022; Yu et al., 2022). The key cause of primary HUA is a combination of low UA excretion and high UA production. A majority (67%) of UA in the human body is produced by the catabolism of nuclear proteins, nucleic acids, and other substances in the body; the remaining 33% comes from purines in food (Lai et al., 2021; Lee et al., 2022; Mccormick et al., 2022). Adenosine deaminase (ADA) and xanthine oxidase (XO) are the key enzymes that regulate the production of UA during the catabolism of purine substances to UA. ADA is a sulfhydryl enzyme that catalyzes the reaction of adenine nucleosides to produce hypoxanthine nucleosides. Hypoxanthine is then produced through the action of nucleoside phosphorylase, and hypoxanthine is finally oxidized by the flavin protease XO to produce UA and XO is a flavin protease (Zhang et al., 2018; Jiang et al., 2020; Le et al., 2020; Michael et al., 2020; Xu et al., 2021).

In previous reports, ten flavonoids belonging to quercetin, isorhamnetin, and kaempferol were detected in the leaves of Changjiang No. 1 (CJ-1) by transcriptomics and metabolomics methods (Tan et al., 2021). Metabolomics and transcriptomics analyses provide us an opportunity of comprehending the flavonoid biosynthesis of the developing seed of Tartary Buckwheat. A total of 234 flavonoids were identified, containing 10 isoflavones, of which 80 flavonoids accumulated prominently in the period of seed development (Li et al., 2019a). The flavonoid biosynthesis of different colored flowers in safflower was analyzed by metabonomics and transcriptomics. Metabolic analysis showed that there are great differences in flavonoid metabolites among different colored safflower (Wang et al., 2021)

Natural bioflavonoids have a small molecular weight and can penetrate adipose tissue, pass through the blood-brain barrier, and are quickly absorbed by the human body these are the material basis for bioflavonoids to play a pharmacological role (Zuo et al., 2012; Yu et al., 2015). In recent years, scholars both domestically and internationally have carried out research into the active components and functional mechanism of such botanical drugs and found that flavonoids such as morin, quercetin, luteolin, and kaempferol have significant effects in treating hyperuricemia (HUA) and gout (Mo et al., 2007; Ouyang et al., 2021). Studies have shown that flavonoids can prevent HUA by inhibiting the activity of xanthine oxidase (XO) and by promoting the excretion of uric acid. Gouty arthritis can be prevented by inhibiting the release of inflammatory transmitters by neutrophils and by inhibiting the expression and secretion of inflammatory cytokines, which are induced by urate crystallization (Martin et al., 2010; Li et al., 2022a). Making full use of Actinidia arguta Sieb. Zucc. flavonoids to develop flavonoid products has broad application prospects in the field of medicine and food homology. At present, the uric acid-reducing activities and biosynthetic pathways of its flavonoids, aspen, rutin, and daidzein, have not been systematically analyzed.

Some scholars analyzed the metabolomes and transcriptomics of the flesh of two kinds of Actinidia arguta Sieb. Zucc. at different fruit development stages, namely “Hong Bao Shi Xing” and “Yong Feng No. 1”. The results showed that AaF3H, AaLDOX, AaUFGT, AaMYB, AabHLH and AaHB2 were the most likely candidate genes to regulate the biosynthesis of flavonoids. Meanwhile, in another study, it was found that the AaLDOX gene may be the key gene controlling anthocyanin biosynthesis in the flesh of “Tian Yuan Hong” Actinidia arguta Sieb. Zucc., which promotes anthocyanin accumulation and eventually leads to red flesh (Li et al., 2018a; Li et al., 2018b). They then screened miR858 involved in anthocyanin biosynthesis through high-throughput sequencing of microRNA and proved miR858 was a negative regulator of anthocyanin biosynthesis by inhibiting the target gene AaMYBC1 in red Actinidia arguta Sieb. Zucc. (Li et al., 2019b; Li et al., 2020).Studies have shown that two interacting transcription factors AcMYB123 and AcbHLH42 and another AcMYB10 have a regulatory effect on the biosynthesis of tissue-specific anthocyanins in the endocarp of Actinidia arguta Sieb. Zucc. (Wang et al., 2019b; Yu et al., 2019).Yanfei Liu et al. showed the cMYBF110-AcbHLH1-AcWDR1 complex directly targeted the promoter of the anthocyanin synthesis gene and promoted the activities of AcMYBF110, AcbHLH1, and AcWDR1. The AcMYBF110-AcbHLH4/5-AcWDR1 complex amplified the regulatory signal of the first MBW complex by activating the promoter of AcbHLH1 and AcWDR1 and indirectly participated in the regulation of anthocyanin synthesis (Liu et al., 2021).

In this study, the uric acid-lowering effect of flavonoid extract from Actinidia arguta Sieb.Zucc. was evaluated *in vitro*. Furthermore, the types, quantities, and differences between flavonoids in the fruits of two important varieties of Actinidia arguta Sieb.Zucc. cultivated in Northern China were determined through a combination of extensive targeted metabolomics and transcriptomics analyses. The biosynthetic pathways and structural genes involved in regulating the flavonoid compounds Chrysin (Flavone1), Rutin (Flavone2), and Daidzein (Flavone3), which have uric acid-reducing activity, were analyzed and identified. This provides valuable information for further improving the fruit quality of Actinidia arguta Sieb.Zucc., breeding new varieties, and developing food and drug homologous functional foods from Actinidia arguta Sieb.Zucc.

## Materials and methods

### Plant Materials and sampling

8-year-old Actinidia arguta Sieb.Zucc. the mature fruit of Qssg and Lc varieties was obtained from North China, which mature from August to September. Fresh fruit was quickly frozen for the next experiment.

95% ethanol, petroleum ether, n-butanol, rutin standard, HPD600 macroporous adsorption resin, and absolute ethanol are all analytical pure. Allopurinol sustained release capsule, purchased from Heilongjiang aolidanede Pharmaceutical Co., Ltd; Ethambutol hydrochloride tablets, purchased from Hangzhou Minsheng Pharmaceutical Co., Ltd; Adenine, purchased from American sigma company; Acetaminophen sustained release tablets, purchased from Shanghai Johnson & Johnson Pharmaceutical Co., Ltd; UA (uric acid) kit, bun (urea nitrogen) kit, Cr (creatinine) kit, GAPDH (glyceraldehyde 3-phosphate dehydrogenase) kit and glycogen kit were purchased from Quanzhou konodi Biotechnology Co., Ltd.

Kunming white mice, weighing 19-21g, were purchased from Shenyang Changsheng Biology Co., Ltd.Before starting the experiment, they were settled in the laboratory environment for seven days. 6 animals for one cage (320× 180 × 160 cm), according to the 12-hour/12-hour light and dark schedule. Temperature: 22 ± 2 °C; Relative humidity: 55 ± 5% and food and water were given in the standard. Our experiments were conducted based on the requirements of the institutional animal care committee of Nanjing University and the China Animal Care Council of Nanjing University [SYSK (SU) 2009 – 0017].

### Flavonoids analysis in two *Actinidia arguta Sieb.Zucc.* varieties

A UPLC-MS/MS analysis conducted by Metware Biotechnology Co., Ltd. (Wuhan, China) detected 786 metabolites. To prepare the biological samples for analysis, they were first freeze-dried in a vacuum freeze dryer (Scientz-100F). Next, the samples were ground (30 Hz, 1.5 minutes) to powder. Then 100 mg of the resulting powder was dissolved in 1.2 ml of 70% methanol extract and vortexed 6 times, once every 30 minutes for 30 seconds, and placed into a 4°C refrigerator overnight. Samples next underwent centrifugation (rotating speed 12000 rpm, 10 minutes) followed by absorption of the resulting supernatant. Finally, the samples were filtered through a 0.22 μM microporous membrane and stored in an injection bottle for UPLC-MS/MS analysis. Using a self-built MWDB (metal database), a qualitative analysis of substances was carried out based on the secondary spectrum information using triple quadrupole multiple reaction monitoring (MRM) mass spectrometry (Fraga et al., 2010). Software Analyst 1.6.3 was used to process mass spectrometry data. We employed principal component analysis to preliminarily explore the general metabolic differences and variabilities between samples. The PCA results display a trend of metabolomics separation between groups, suggesting metabolomics differences between sample groups (Chen et al., 2009). The metabolomics data were analyzed according to the OPLS-DA model, and score maps were drawn to further show the differences between each group (∣log2 (fold change) ∣ ≥ 1) (Thévenot et al., 2015). Metabolites in each sample were analyzed, with three independent biological replicates.

### Flavonoid isolation, identification, and uric acid-lowering activity test

Take the frozen Actinidia arguta Sieb.Zucc. and wash it with distilled water after melting. Dry it in the air, slice it, and grind it in a homogenizer until it is homogenized. After ethanol extraction, centrifugation, filtration, and concentration, the crude extract of total flavonoids of Actinidia arguta Sieb.Zucc. was obtained, which was used for standby.

A Rutin standard curve was generated to determine the concentration of flavonoids in the crude extract. Macroporous resin and preparative liquid chromatography were then used for separation and purification. In this experiment, flavonoids were identified by electrospray spray mass spectrometry.

Thirty-six male Kunming mice were separated into six groups at random after seven-day adaptive feeding in the laboratory environment at 22 ± 2°C and a 55 ± 5% relative humidity. The groups were as follows: blank control group, model control group, positive control group (Allopurinol), Chrysin(Flavone1), Rutin (Flavone2), and Daidzein (Flavone3). The blank group was gavaged with distilled water, and the animals in all other groups were gavaged with a 2.5% PAPA suspension for seven consecutive days. Then the mouse model of hyperuricemia was induced by gavage with 100 mg/kg adenine and 250 mg/kg ethambutol. Animals in the treatment groups (i.e., groups other than the blank and model controls) were given the same volume of distilled water, and the appropriate drugs were administered by gavage. The dose of flavonoids was 550 mg/kg. The dosage of allopurinol tablets was 33.3 mg/kg (administration volume: 1 ml/100 g) for 23 consecutive days.

One hour after treatment administration on the 7th and 15th days, eyeball blood was collected. The serum was centrifuged and serum levels of uric acid (UA), urea nitrogen (BUN), creatinine (Cr), glyceraldehyde 3-phosphate dehydrogenase (GAPDH), and hepatic glycogen were measured using kits.

### RNA extraction, library construction, and sequencing

mRNA with PolyA tail was enriched using Oligo (dT) magnetic beads, and then chemically fragmented. Using the resulting short segment RNA as a template, the first strand of cDNA was synthesized with six base random primers/hexamers. Subsequently, the double-stranded cDNA was synthesized by adding buffer, dNTPs, and DNA polymerase I, and purified with ampure XP beads, and then was subjected to end repair, A-tail addition, and connect sequencing connector, and fragment size was selected with ampure XP beads. PCR enrichment yielded the final cDNA library. Qubit2.0 was used for preliminary quantification, Agilent 2100 was used to detect the insert size of the library, and the Q-PCR method quantified the effective concentration of the library (> 2nm). After passing the library inspection, the libraries were pooled according to the target offline data volume, and Biomarker Technology Co., Ltd. (Beijing, China) conducted the sequencing using the Illumina novaseq platform.

### RNA sequencing data analysis

Clean reads for subsequent analysis were obtained following raw data filtering, sequencing error rate inspection, and GC content distribution inspection. The clean reads were spliced with Trinity (Grabherr et al., 2011), and stored in FASTA format. Unigene corset (Davidson and Oshlack, 2014) hierarchical clustering was used to obtain the longest cluster sequence, which was then compared with the KEGG, NR, Swiss-Prot, GO, COG/KOG, and Trembl databases using DIAMOND (Buchfink et al., 2015) BLASTX software. After predicting the amino acid sequence, HMMER software was used to compare the sequence with the Pfam database to obtain the Unigene annotation information. RSEM (Li and Dewey, 2011) software and bowtie2 (Langmead and Salzberg, 2012) were used to compare the statistical results. FPKM (fragments per kilobase of transcription per million fragments mapped) was taken as an index to detect the level of transcripts or gene expression. DESeq2 (Love et al., 2014; Varet et al., 2015) was used to obtain the differential expression between the two biological conditions. The FDR (false discovery rate) value was <0.05 and ∣ log2 (folding change) ∣ ≥ 1 was used as the threshold of significant expression difference. Through GOannotation and KEGG pathway analysis, the identified DEG was further enriched and analyzed.

### qRT-PCR

To validate the RNA-Seq data and examine the expression of flavonoid biosynthesis-related genes, qRT-PCR was carried out as described in the previous literature. The amplification cycle procedure was as follows: the reverse transcription operation was carried out using an Aidlab company’s kit (TUREscript 1st Stand cDNA SYNTHESIS Kit), and the 20ul reaction system was adopted. The reverse transcription reaction conditions were 42 °C for 40min and 65 °C for 10min, and the fluorescent quantitative PCR procedure was 95 °C for 3min, 95 °C for 10s, and 60 °C for the 30s. The relative gene expression of each sample and group was calculated by using 2^-△△Ct^ with actin as the internal reference. Each sample was performed in triplicates. **Table S1** lists the primers used in qRT-PCR.

### Correlation analysis between metabolites and transcripts

The correlation coefficient was calculated for the content of flavonoids and the transcriptional changes of both differentially expressed flavonoids and differentially expressed genes. Both are rich in biosynthesis pathways of flavonoids, the flavonol (ko00941), flavonoid (ko00942), and secondary metabolite (ko00943). Cytoscape2.8 was used to visualize the interaction network between DEGs and differentially accumulated flavonoids to identify the structural genes involved in the regulation of flavonoids, such as Chrysin (Flavone1), Rutin (Flavone2), and Daidzein (Flavone3), with uric acid reducing activity.

## Results and analysis

### Flavonoid composition and uric acid-reducing capacity of two varieties of *Actinidia arguta Sieb. Zucc*.

**Figure 1:**
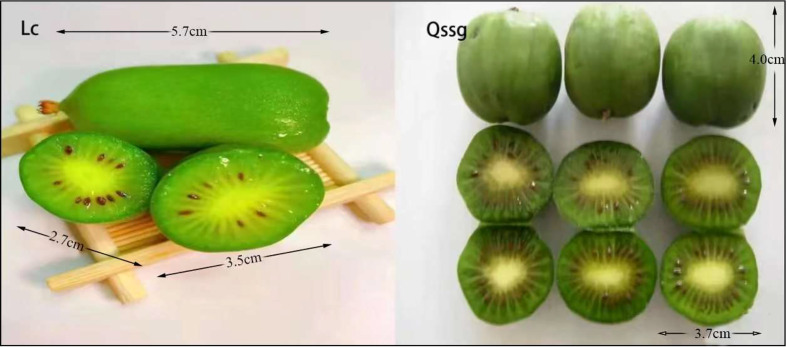
Qssg and Lc of two main cultivars of *Actinidia arguta Sieb.Zucc*.in Northern China.

**Figure 2:**
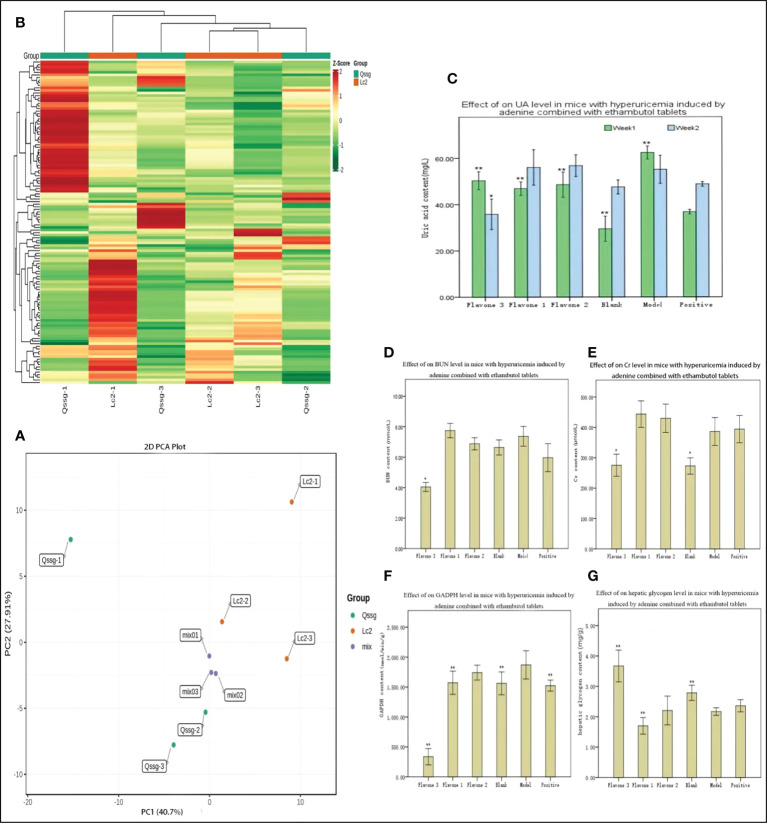
Composition of flavonoids and uric acid reducing analysis results in Qssg and Lc *Actinidia agruta Sieb.Zucc*. **(A)** PCA score map of metabolites in Qssg and Lc fruits, each point representing an independent biological repeat. **(B)** Clustering heat map of metabolites in Qssg and Lc fruits. There are obvious differences between the two samples. **(C)** Effect of adenine combined with UA levels in mice with hyperuricemia. **(D)** Effect of adenine combined with ethambutol tablets and acetaminophen on BUN levels in mice with hyperuricemia. **(E)** Effect of adenine combined with ethambutol tablets and acetaminophen on Cr levels in mice with hyperuricemia. **(F)** Effect of adenine combined with ethambutol GAPDH levels in mice with hyperuricemia. **(G)** Effect of adenine combined with tablets and acetaminophen on hepatic glycogen levels in mice with hyperuricemia. "*" Indicates significant, “**” indicates extremely significant.

A total of 125 flavonoids were identified by qualitative and quantitative analysis of the Qssg and Lc metabolite spectrum. The mature fruits of Qssg and Lc are shown in **Figure 1** (**Table S2**). PCA revealed these two varieties to be significantly different; 68.61% of the differences between the samples could be explained by PC1 (40.7%) and PC2 (27.91%), suggesting their pattern change of flavonoid accumulation (**Figure 2A**). Hierarchical cluster analysis (HCA) further confirmed the difference between the two main samples (**Figure 2B**). Chrysin (Flavone1), Rutin (Flavone2), and Daidzein (Flavone3) in both Qssg and Lc fruits were isolated and purified. A male Kunming mouse animal model was used to carry out a uric acid lowering activity test (**Figures 2C–G**).

Compared with the model control group, the three selected flavonoids significantly reduced UA after one week (p < 0.01) (**Figure 2C**). At two weeks, Daidzein significantly reduced UA (p < 0.05), but there was no significant difference in UA activity between Chrysin and Rutin. Daidzein had relatively stable biological activity in reducing UA. Compared with the blank control group, BUN was not significantly increased in the model control group (**Figure 2D**). Compared with the model control group, only Daidzein significantly reduced BUN (p < 0.05), and there was no significant difference in the BUN-reducing activity of the other two flavonoids. Thus, Daidzein had a higher biological capacity to reduce BUN than Chrysin or Rutin did. Daidzein also significantly reduced Cr compared to the model control group (p < 0.05), but there was no significant difference in Cr reducing activity between the other two Flavone treatment groups (**Figure 2E**). Compared with the model control group, Rutin, Daidzein, and the positive control group (treated with allopurinol) all had significantly lower levels of GAPDH activity (p < 0.01), meaning that only the Chrysin treatment group showed no significant difference (**Figure 2F**). Daidzein also significantly increased liver glycogen compared to the model control group (p < 0.01); in contrast, Rutin significantly decreased liver glycogen (p < 0.01), and there was no significant difference in liver glycogen in those treated with Chrysin (**Figure 2G**).

### Differences of flavonoids in Qssg and Lc fruits, enrichment analysis of KEGG pathway, and structural verification results

Orthogonal signal correction (OSC) and PLS-DA techniques were used to identify variable differences. Abundances between samples of the 125 flavonoids were compared using an OPLS-DA model. The model was able to discriminate between the two varieties as both samples fell outside the confidence interval, with the Lc samples to the left and the Qssg samples to the right of the interval.

**Figure 3:**
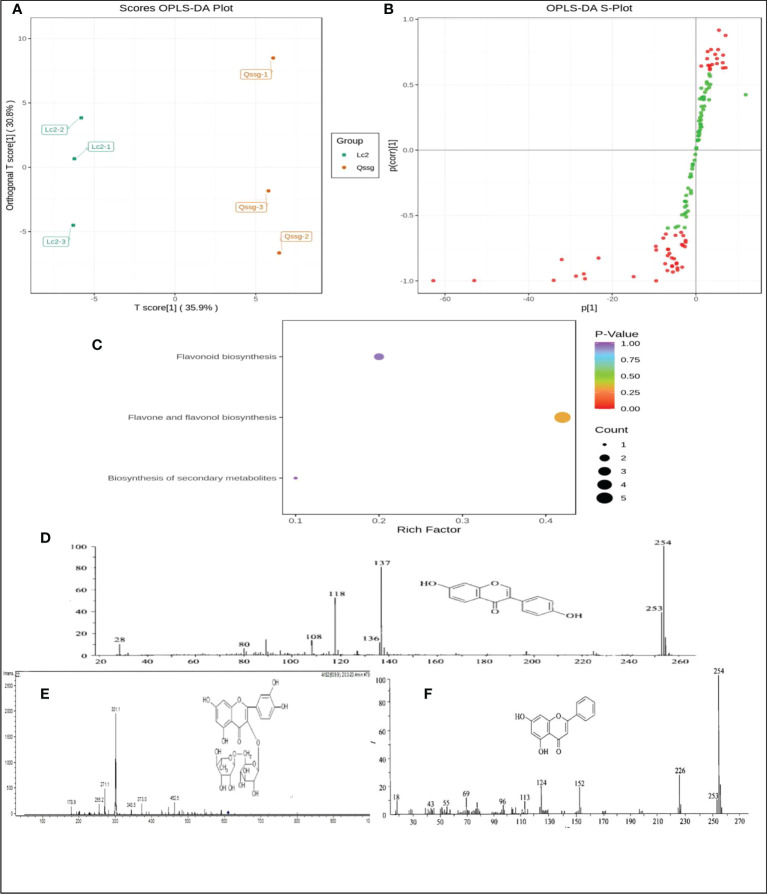
Difference of flavonoids in Qssg ang Lc *Actinidia arguta Sieb.Zucc*., enrichment analysis results of KEGG pathway and structure diagram of Rutin, Chrysin and Daidzein. **(A)** Opls-da score map of metabolites in Qssg and Lc fruits, and each point represents an independent biological repeat. **(B)** OPLS-DA S-plot of metabolites in Qssg and Lc fruits(p<0.05).**(C)** KEGG classification map of differential metabolites in Qssg and Lc fruits. **(D)** Rutin ionization and mass spectrum.e ionization and mass spectrum of populin. **(E)** Chrysin ionization and mass spectrum. **(F)** Daidzein ionization and mass spectrometry.

The OPLS-DA yielded two principal components with contribution rates of 62.1% and 9.74% (R^2^x = 0.807, R^2^y = 0.998 [p = 0.29], Q2 = 0.869 [p < 0.005]). This result was verified by 200 replicate analyses. The differential flavonoids were screened according to VIP analysis (**Figures 3A, B**).

The pathway enrichment analysis of flavonoids was carried out through KEGG (Kyoto Encyclopedia of genes and genes) database. The results showed that the 125 identified flavonoids were mainly distributed in three metabolic pathways,(1) flavonoid and flavonol biosynthesis pathways, primarily for kaempferol-3-o-neohesperidin Luteolin-7-o-neohesperidin, quercetin-3-o-sangbu diglycoside, quercetin-3-o-(2”-o-xylosyl)-rutoside, and kaempferol-3-o-rutoside;(2) flavonoid biosynthesis pathways, mainly including naringin-7-o-glucoside, isolyceride, and chrysin;(3) secondary metabolite biosynthesis pathways, primarily kaempferol-3-o-rutoside and daidzein (**Figure 3C**).

In a mass spectrum graph, the mass charge ratio (M/z) of the ion increases from left to right, and the abscissa of an ion with a single charge is the mass of its ion; the ordinate represents the intensity of the ion current, usually expressed in terms of relative intensity. The elution rate is 68.8% for 50% ethanol, 51.2% for 60% ethanol, and 48.2% for 70% ethanol. Based on our test data, the peak value of flavonoids eluted by 50% ethanol was more and the elution rate was higher. Therefore, only positive ion mode mass spectra of flavonoids eluted by 50% ethanol are discussed here. The mass spectrum information for full scanning mode was m/Z 609, 301, and 175, the multi-reaction detection mode was m/Z [609/301], and the neutral loss was 308u. By comparing the retention times, multi-stage mass spectrum fragments, excimer ion peaks, and other information with reference substances, we determined that the molecular ion peak was m/z 609 for Rutin, m/z 254 for Chrysin, and m/z 254 for soybean isoflavone (**Figures 3D–F**).

### Transcriptome analysis of two *Actinidia arguta Sieb.Zucc.* varieties

Nine cDNA libraries were generated for high-throughput RNA-Seq analysis to further examine the potential molecular mechanisms of flavonoid biosynthesis in Actinidia arguta Sieb.Zucc. Each library obtained 281,758,296 clean reads from 439,776,908 to 43,580,384 and 269,732,354 from 41,844,326 to 41,708,692 (**Table S3**). The Q30 percentage (including sequences having an error rate < 0.1%) for each library exceeded 91%, with 47.47% GC content on average. Among the clean reads, between 78.54% and 80.76% could be mapped to the reference genome. A total of 43,686 unique genes wereidentified, supplying high-quality RNA-Seq data for further analyses

### Differential gene expression in two *Actinidia arguta Sieb.Zucc.* varieties

**Figure 4:**
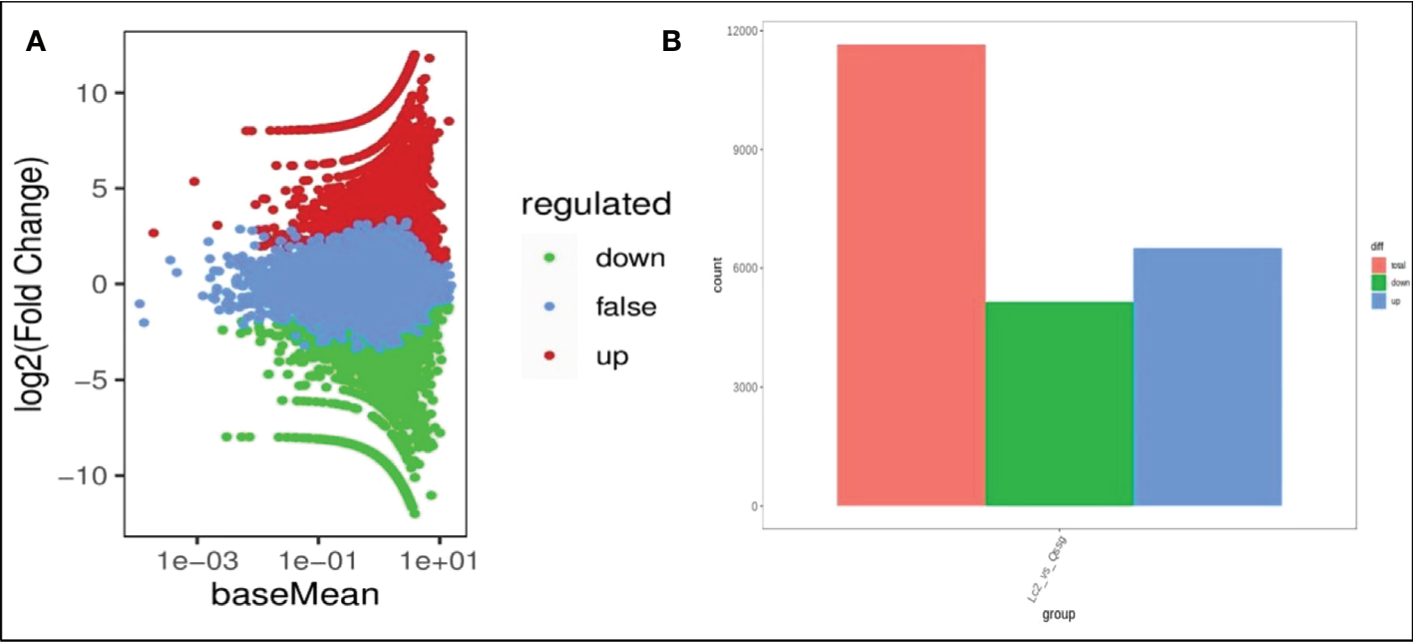
Differential gene expression between Qssg and Lc *Actinidia arguta Sieb.Zucc*.. **(A)** MA map of differential genes between Qssg and Lc fruits. **(B)** Columnar chart of the differential gene number in Qssg and Lc fruits.

**Figure 5:**
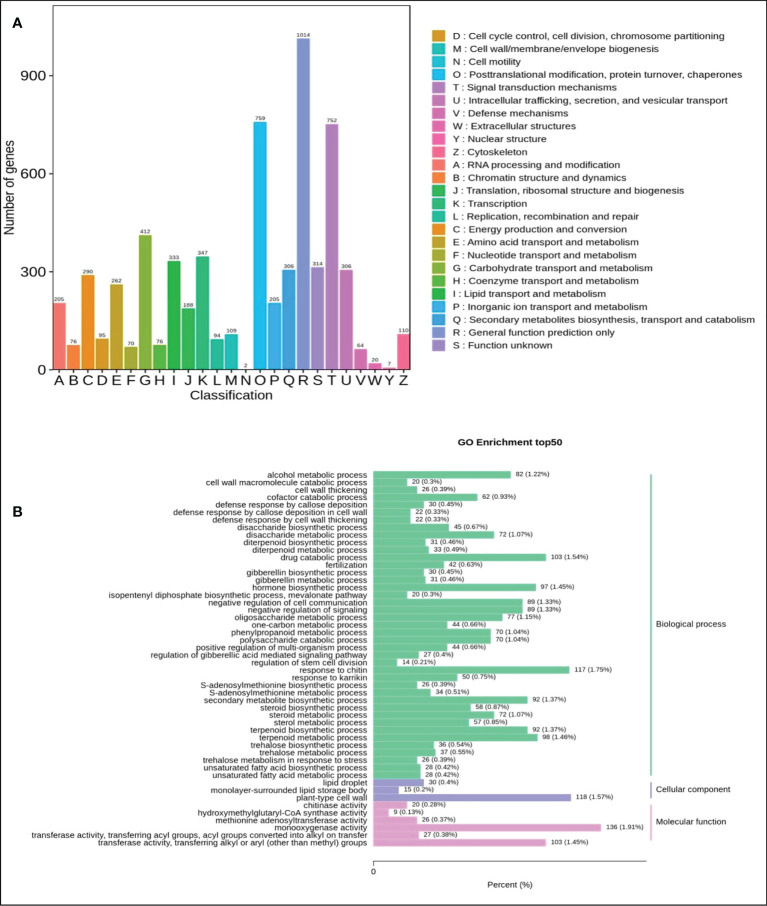
Classification of secondary entries of Qssg and Lc fruit difference genes. **(A)** Column chart of quantitative statistics of Qssg and Lc KOG functional classification. **(B)** Statistical map of the number of differential genes contained in the secondary go entries of Qssg and Lc.

To identify the DEGs in the mature fruits of two kinds of *Actinidia arguta* Sieb.Zucc., the correlation coefficient between gene expression profile clustering and biological duplication was first analyzed (**Figure 4A**), indicating there was a large number of differential expressions between different samples. The gene expression correlation coefficient level between biological replication of all samples were greater than 0.8, indicating that biological replication is very good, and the data can be further used to determine DEG. According to the FDR (false discovery rate) values <0.05 and ∣ log2 (fold change) ∣ ≥ 1 between the two sample groups as the threshold of significantly different gene expression, it was determined that 6497 was up-regulated and 5153 was downregulated between the two sample groups (**Figure 4B**). The most abundant items among the 25 biological process categories were metabolic processes, cellular processes, and single biological processes. The most representative terms among the 13 cell component categories were cell part, cell, and organelle. Among the 10 molecular functional categories, the most common terms were catalytic activity, binding, and transporter activity (**Figure 5A** and **Table S4**).

4024 DEGs were allocated to 142 KEGG paths (**Table S5**). The flavonoid (ko00941), flavonol (ko00944), and secondary metabolite (ko01110) biosynthesis pathways were all enriched, with the flavonoid and secondary metabolite pathways being significantly enriched. Flavonoid biosynthesis and secondary metabolite biosynthesis are present in the significant enrichment pathway (**Figure 5B** and **Table S6**). The enriched pathways could be further divided into five categories: cellular processes, genetic information processing, environmental information, metabolism, and tissue systems. The metabolism category contained the most pathways and the highest number of DEGs were involved in amino acid biosynthesis (ko01230; 185 genes), carbon metabolism (ko01200; 190 genes), and sucrose and starch metabolism (ko00500; 207 genes).

### Regulatory analysis of flavonoid biosynthesis and differential gene expression in two kinds of mature *Actinidia arguta Sieb. Zucc.*

KEGG analysis and gene functional annotations were two strategies to determine which DEGs encode enzymes related to flavonoid biosynthesis, flavonoid and flavonol biosynthesis, and secondary metabolite biosynthesis. The results showed that 50 DEG genes, including 6 UGT9491 genes, 16 LOC genes, 2 AT2 genes, 2 CHS, 3 C4Ha genes, 1 HCT gene, 1 CCoAOMT gene, 1 F3H gene, 2 LAR2 genes, 2 4CL, 1 VIT, 4 PAL, and 1 GSCOC gene, were significantly up-regulated in the two varieties, 2 CFOL genes, 1 CHIa gene, 1 C4Ha gene, 1 DFR gene, 1 LAR2 gene,1 LOC gene, and 1 LSAT gene were significantly down-regulated in the two varieties (**Table S7**).

### qRT PCR was used to confirm the results of DEGs related to flavonoid biosynthesis

**Figure 6:**
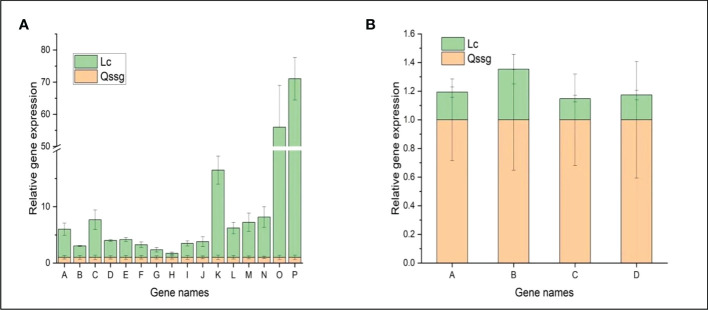
qRT PCR confirmation map of the differential flavonoid-related gene expression in Qssg and Lc *Actinidia arguta Sieb.Zucc.* fruits. **(A)** The relative expression levels of 16 structural genes regulating flavonoids in Lc varieties were higher than those in Qssg varieties. **(B)** The relative expression levels of the four structural genes regulating flavonoids in Lc varieties were lower than those in Qssg varieties. Most of the selected genes showed a high correlation between the qPCR and RNA-seq datasets, thus validating the transcriptome data. ( 2^-△△Ct data value of the internal reference gene is ACTIN).

To test the expression of DEGs related to flavonoid biosynthesis in the fruits of the two varieties at maturity, Twenty structural genes (1 CsUGT134, 1 LAR2, 2 C4H, 1 CFOL, 1 CHI, 2 LOC, 1 GSCOC, 1 CCoAOMT, 1 DFR, 1 AT2,2 4CL,1 VIT,4 PAL,1 CHS) were quantified by qRT-PCR. The detected genes were highly consistent between the RNA-Seq and qPCR results according to Reverse transcriptase polymerase chain reaction results(**Figure 6**). The relative expression of CFOL, CsUGT134, PAL, C4Ha, CHS, and LOC in Lc was significantly higher than that in Qssg; the relative expression of CHIa, LAR2, GSCOC, and CCoAOMT in regulating flavone content in Qssg varieties was significantly higher than that in Lc varieties. The result confirmed the transcriptomics-derived data.

### Correlation between transcripts and flavonoid derivatives

**Figure 7:**
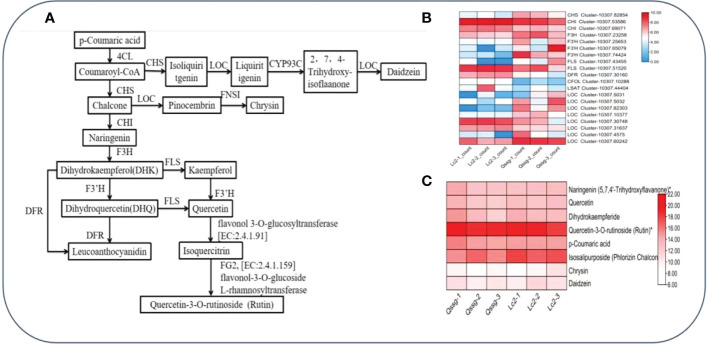
Flavonoid biosynthesis pathway.Metabolite content and regulatory structure gene network in Qssg and Lc fruits. **(A)** Biosynthetic pathway of Rutin, Chrysin, and Daidzein in Qssg and Lc fruits. **(B)** Determination of gene expression in Rutin, Chrysin, and Daidzein biosynthesis pathway in Qssg and Lc fruits. **(C)** Determination of metabolites in Rutin, Chrysin, and Daidzein biosynthesis pathway of Qssg and Lc fruits.

To comprehend the pathway and regulatory structural genes of flavonoid biosynthesis in the two main varieties of *Actinidia arguta* Sieb.Zucc. fruit, the quantitative changes of flavonoids and transcripts in the fruit ripening stage of *Actinidia arguta* Sieb.Zucc. were tested and analyzed by studying the interaction between transcriptomics and metabolomics. Based on the results of DEGs and Dems rich in flavonoid biosynthesis pathway, it was annotated that 20 structural genes and regulatory groups show a higher correlation with the biosynthesis pathway of flavonoid compounds Chrysin, Rutin, and Daidzein (**Table S8**). Their interaction network is shown in (**Figures 7A–C**). According to the analysis of the pathway diagram in **Figure 7A**, DFR, F3’H, and FLS compete with the substrate dihydrokaempferol DHK. According to the gene expression analysis in **Figure 7C**, FLS has relatively high expression and high activity of its coding enzyme, which boosts the synthesis of Rutin. It was also found that the relative content of Rutin accumulated in the sample was high (**Figure 7B**). The LOC expression was comparatively low, and the accumulated Chrysin and Daidzein substances were relatively low.

According to the analysis in the pathway diagram in **Figure 7A**, DFR, F3’H, and FLS compete with the substrate dihydrokaempferol DHK. According to the gene expression analysis in **Figure 7C**, FLS has relatively high expression and high activity of its coding enzyme, which finally promotes the synthesis of Rutin. The relative content of Rutin accumulated in the sample was high (**Figure 7B**); the expression of LOC and the relative contents of Chrysin and Daidzein was relatively low.

## Discussion

Flavonoids are substances that have been studied in depth and at length in traditional Chinese medicine. They exist widely throughout the kingdom Plantae. Many studies have focused on the abilities of flavonoids to reduce blood lipid levels, inhibit lipid peroxidation, relieve coughs, eliminate phlegm and asthma, and exert anti-tumor, anti-hepatotoxicity, anti-inflammatory, analgesic, antibacterial, and antispasmodic effects. Substances known to reduce uric acid include quercetin, luteolin, apigenin, puerarin, catechins, and dyestuffs, but there have been few studies into the uric acid-lowering activities of Rutin, Chrysin, and Daidzein.

In this study, extensive targeted metabolomics methods were used to compare the flavonoids present in the fruit of two varieties of Actinidia arguta Sieb.Zucc. cultivated in Northern China. 9 classes and 125 kinds of flavonoids were detected in the fruits of the two varieties; these included 39 kinds of differentially accumulated flavonoids, accounting for 31.2% of the total flavonoids detected. This demonstrated that there were significant differences in flavonoid composition and accumulation between different varieties. The types and proportions of different flavonoids found in the fruits were as follows: 51.2% flavonols, 14.4% flavonoids, 9.6% dihydroflavonoids, 9.6% flavonols, 1.6% chalcones, 2.4% flavone carboglycosides, 4.8% dihydroflovonols, 0.8% isoflavones, and 5.6% procyanidins. They are mainly distributed in 3 metabolic pathways, among which the flavonoid and flavonol biosynthesis pathways mainly include kaempferol-3-O-neohesperidin, luteolin-7-O-neohesperidin, quercetin- 3-O-Sambubiglycoside, quercetin-3-O-(2”-O-xylosyl)rutinoside, kaempferol-3-O-rutinoside; quercetin-3-O-rutinoside (Rutin); the flavonoid biosynthesis pathway mainly includes naringenin-7-O-glucoside, isoflavin, and Chrysin; the secondary metabolite biosynthesis pathway mainly includes kaempferol-3-O-rutinoside, Daidzein. The relative content of flavonoids in the fruit of the variety Lc was increased by comparing it with the fruit of the variety Qssg Actinidia arguta Sieb. Zucc.These data provide a strong basis for the enrichment study of flavonoids in Actinidia arguta Sieb. Zucc. and also provide a valuable theoretical and practical basis for breeding new varieties of Actinidia arguta Sieb.Zucc. and developing food and drug homologous functional foods.

The flavonoids Rutin, Chrysin, and Daidzein were isolated from the fruit of the main variety of Actinidia arguta Sieb. Zucc. cultivated in northern China and tested for their uric acid-reducing activity. The results showed that Chrysin, Rutin, and Daidzein could reduce serum levels of UA, BUN, Cr, and GAPDH in mice to varying degrees. We, therefore, speculate that uric acid production in mice (in response to purine-rich food ingestion or catabolism of substances such as nuclear proteins and nucleic acids) may be inhibited by reducing the activities of ADA and/or XO. Increases in liver glycogen content in mice may be due to inhibition of glycolysis by flavonoids through the promotion of gluconeogenesis, reduction of free glucose in the blood, promotion of the remedial synthesis pathway of nucleotides, and decreases in serum uric acid content. This study, therefore, has strong practical significance for the prevention and control of human HUA through the comprehensive development of Actinidia arguta Sieb.Zucc. as a treatment.

There are two general kinds of genes involved in the biosynthesis of plant flavonoids: structural genes, which encode enzymes that catalyze the flavonoid biosynthesis, and regulatory genes, which regulate the structural genes’ expression levels (Gupta et al., 2011; Li et al., 2012; Chen et al., 2017). Transcriptomics analysis identified 43,686 genes involved in flavonoid biosynthesis in the fruits of two Actinidia arguta Sieb. Zucc. varieties, including 11,650 differentially expressed genes. These genes regulate metabolism, cellular processes, genetic information processing, environmental information, and tissue systems in the Actinidia arguta Sieb. Zucc. fruit. KEGG enrichment analysis and gene function annotation identified 20 structural genes encoding 50 known flavonoid biosynthesis-related enzymes, with most of the genes highly correlated between the qPCR and RNA-seq datasets.

The correlation analysis between the transcriptomics and metabolic spectrum revealed the expression level of some structural genes to be bound up with the accumulation of particular flavonoids, indicating that the expression of these flavonoid biosynthesis genes contributed to the accumulation of flavonoids in the fruit ripening of the two main Actinidia arguta Sieb. Zucc. cultivars. Across many plants, naringin, kaempferol, kaempferol, myricetin, dihydromyricetin, and dihydro quercetin are catalyzed by positively regulating the expression of CHS, CHI, F3H, F3’H, and FLS (Liu et al., 2002; Deavours and Dixon, 2005; Liu et al., 2016; Wang et al., 2017; Matsui et al., 2018; Wang et al., 2019a; Jin et al., 2022; Li et al., 2022b). In addition, CHS, CHI, F3H, CFoL, LOC, LSAT, FNSI, DFR, F3’H, FLS, and HIDH may play structural or regulatory roles in the biosynthetic pathways of the flavonoid compounds chrysin (Flavone1), rutin (Flavone2) and daidzein (Flavone3). DFR, F3’H, and FLS compete with the substrate dihydrokaempferol (DHK). FLS expression and the encoded enzyme activity were high, which ultimately promotes Rutin accumulation. LOC expression and the levels of Chrysin and Daidzein were relatively low.

In conclusion, the research results will continue to expand our further development of Actinidia arguta Sieb. Zucc. resources in northern China, and will further promote our exploration of the molecular basis of using flavonoids in Actinidia arguta Sieb. Zucc. to prevent and control hyperuricemia and its biosynthesis.

## Data availability statement

The data presented in the study are deposited in the NCBI repository, accession number PRJNA649743.

## Ethics statement

The animal study was reviewed and approved by Shenyang Agricultural University Institutional Animal Care and Use Committee (IACUC).

## Author contributions

LC and YW designed and drafted the manuscript. YBW performed experiments and analyzed the data. YBW wrote the first draft of the manuscript with the help of KD and YW. XY has been involved in partial data analysis and figure compile and edit. MZ, CH and MI helped to review and edit the manuscript. LC has been involved in critically revising the manuscript for important intellectual content. All authors contributed to the article and approved the submitted version.

## Conflict of interest

The authors declare that the research was conducted in the absence of any commercial or financial relationships that could be construed as a potential conflict of interest.

## Publisher’s note

All claims expressed in this article are solely those of the authors and do not necessarily represent those of their affiliated organizations, or those of the publisher, the editors and the reviewers. Any product that may be evaluated in this article, or claim that may be made by its manufacturer, is not guaranteed or endorsed by the publisher.
